# An air-locking port and high-flow nasal cannula in non-intubated uniportal video-assisted thoracic surgery for pneumothorax with pulmonary dysfunction: a case report

**DOI:** 10.1186/s40792-021-01321-5

**Published:** 2021-10-26

**Authors:** Hiroya Yamagishi, Yusuke Wakatsuki, Toshihiko Tada, Tadashi Matsukura

**Affiliations:** 1 Department of Chest Surgery, Japanese Red Cross Fukui Hospital, 2‑4‑1 Tsukimi, Fukui, Fukui 918‑8501 Japan; 2 Department of Thoracic Surgery, Kyoto University Graduate School of Medicine, 54 Kawahara-cho, Shogoin, Sakyo-ku, Kyoto, 606-8507 Japan; 3 Department of Respiratory Medicine, Japanese Red Cross Fukui Hospital, 2‑4‑1 Tsukimi, Fukui, Fukui 918‑8501 Japan

**Keywords:** Secondary spontaneous pneumothorax, Interstitial pneumonitis, Idiopathic pulmonary fibrosis, Non-intubated video-assisted thoracic surgery, Uniportal video-assisted thoracic surgery, Single-incision surgery, Air-locking port, High-flow nasal cannula, Surgical pneumothorax

## Abstract

**Background:**

Non-intubated video-assisted thoracic surgery is a therapeutic option for intractable secondary spontaneous pneumothorax in patients who are poor candidates for surgery with endotracheal intubation under general anesthesia. However, intraoperative respiratory management in this surgery is often challenging because of hypoxia caused by surgical pneumothorax.

**Case presentation:**

A 75-year-old man with idiopathic pulmonary fibrosis who had been on home oxygen therapy underwent non-intubated uniportal video-assisted thoracic surgery for intractable spontaneous pneumothorax. During the operation, oxygen was administered using a high-flow nasal cannula at a high flow rate. An air-locking port for single-incision surgery was used to minimize the inflow of air into the pleural cavity. The intrapleural air was continuously suctioned through the chest tube. The air-leak point was easily identified and closed using ligation. Oxygenation was satisfactory throughout the operation.

**Conclusions:**

Non-intubated uniportal video-assisted thoracic surgery for secondary spontaneous pneumothorax with an air-locking port, continuous pleural suction, and high-flow nasal cannula may achieve satisfactory intraoperative oxygenation in patients with respiratory dysfunction. The intrapleural space can be feasible for surgical manipulation without surgical pneumothorax in non-intubated video-assisted thoracic surgery even when supplied with oxygen at a high flow rate using a high-flow nasal cannula.

## Background

Pneumothorax with prolonged air leakage is an indication for surgical intervention. Patients with secondary spontaneous pneumothorax (SSP) include poor candidates for surgery with endotracheal intubation under general anesthesia due to various conditions, including impaired cardiopulmonary reserve [1]. In these cases, video-assisted thoracic surgery (VATS) without endotracheal intubation or non-intubated VATS can be a therapeutic option [1–3]. However, respiratory management during non-intubated VATS, especially in patients with impaired respiratory function, is often challenging due to hypoxia caused by surgical pneumothorax [1, 2, 4], in which the air enters the pleural cavity through the opened chest wall. Here, we report a case of SSP with severe respiratory dysfunction safely treated with non-intubated uniportal VATS with an air-locking port and high-flow nasal cannula (HFNC) with satisfactory oxygenation throughout the operation.

## Case presentation

**Fig. 1:**
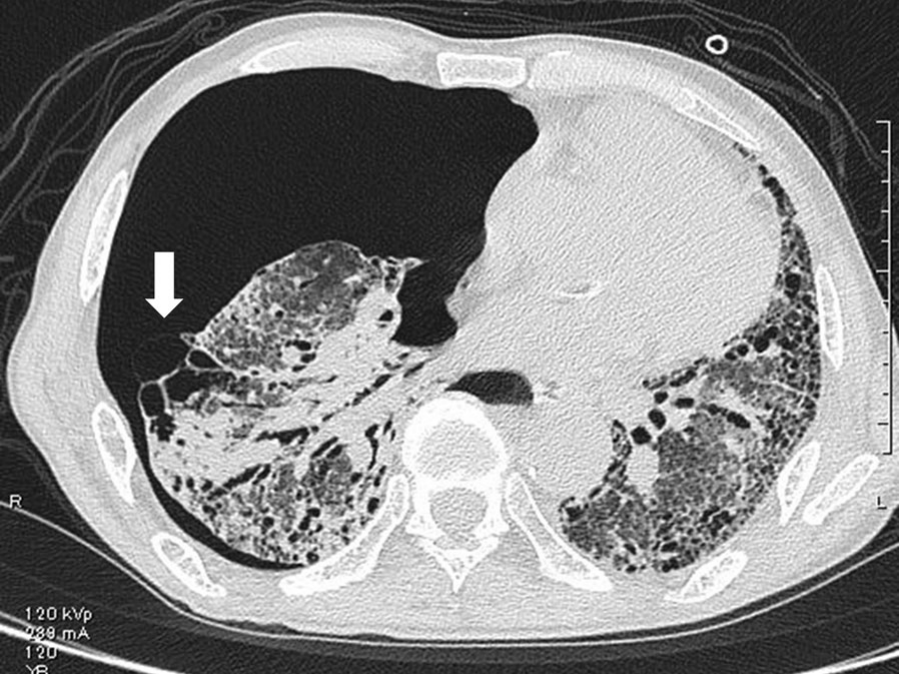
Computed tomography at the onset of pneumothorax showing cystic lesions in the fissure between the right middle and lower lobes (arrow)

**Fig. 2:**
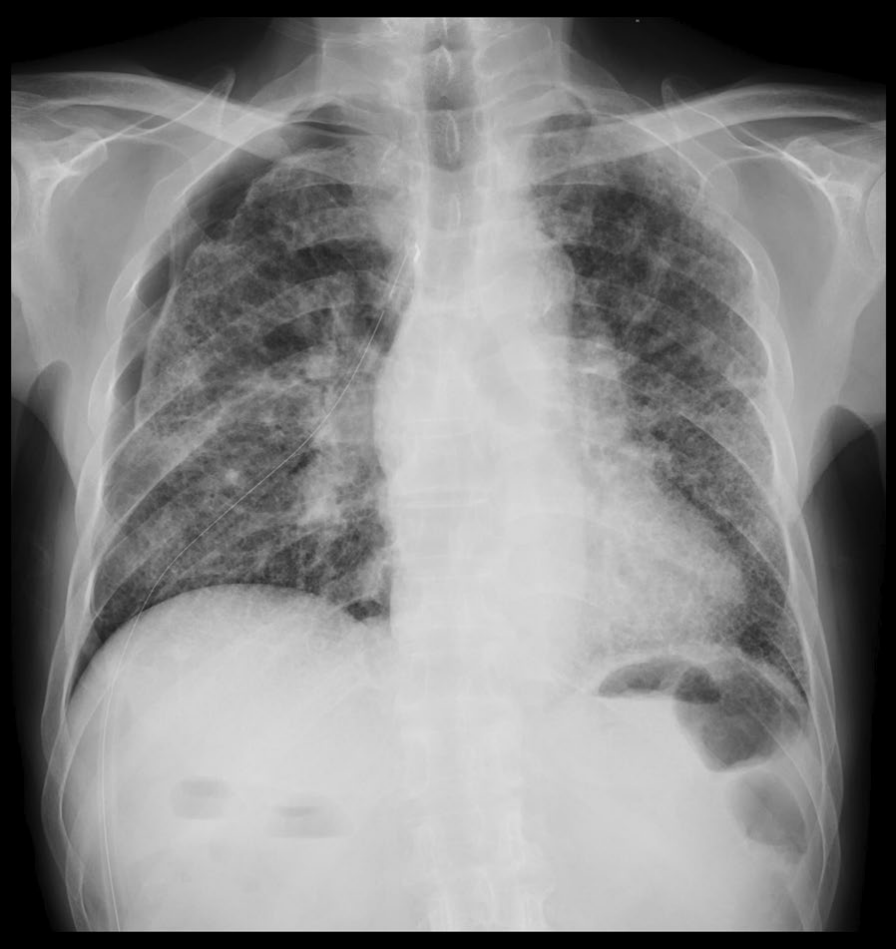
Preoperative chest X-ray showing incomplete expansion of the right lung

**Fig. 3:**
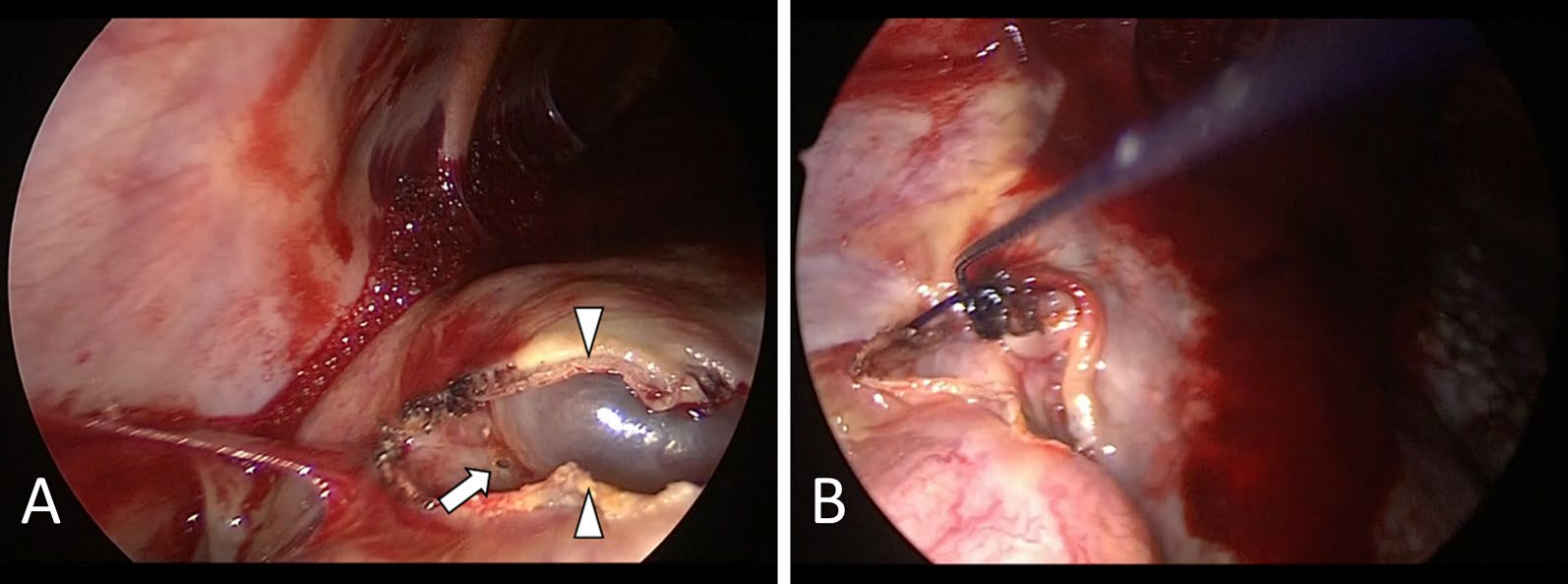
Thoracoscopic images. **A** Image showing a small pore on a cyst in the lower lobe (arrow). Arrowheads indicate edges of the dissected adhesive tissue. **B** Image taken after closure of the air-leak point

A 75-year-old man with idiopathic pulmonary fibrosis who had been on home oxygen therapy was referred to our department for the treatment of right spontaneous pneumothorax. Chest computed tomography showed cystic lesions in the fissure between the right middle and lower lobes (Fig. 1). Chest tube drainage was immediately performed. Although he underwent pleurodesis with autologous blood, moderate air leakage persisted and lung expansion was incomplete (Fig. 2). His oxygenation deteriorated due to pneumothorax. Percutaneous oxygen saturation was 96% with an oxygen conserving device, Oxymizer F-224 (CHAD Therapeutics, California, USA), with oxygen at 6 L/min. Non-intubated VATS under local anesthesia was performed 8 days after the onset of pneumothorax. He was placed in the supine position while breathing spontaneously. Oxygen was administered via an HFNC (Optiflow, Fisher & Paykel Healthcare, Auckland, New Zealand) with 60% inspired oxygen at a flow rate of 60 L/min. Fentanyl and midazolam were administered intravenously for analgesia and mild sedation, respectively. After local anesthesia using 1% lidocaine, an air-locking port for single-incision surgery, GelPOINT Mini (Applied Medical, California, USA), was positioned in a 3-cm incision in the fifth intercostal space at the anterior axillary line. Three 10-mm sleeves were attached to the port. The chest tube was not removed, and intrapleural air was continuously suctioned with a negative pressure of 5 cmH_2_O during the operation. A 5-mm 30-degree rigid video thoracoscope was introduced into the pleural cavity. The adhesive tissue between the middle and lower lobes fluttered up and down as the patient breathed. Dissection of the adhesive tissue revealed a small pore on the cyst in the lower lobe (Fig. 3A). The saline drip confirmed that air leaked from the pore. The diameter of the pore was below 1 mm. The pore was closed by ligation (Fig. 3B) and covered with a polyglycolic acid sheet and fibrin glue. The operating time was 40 min. Percutaneous oxygen saturation level was 95–96% during surgery with constant flow through the HFNC. Surgical manipulation was not interrupted to wait for oxygenation recovery. The pleural suction pressure was maintained at 5 cmH_2_O during surgery. The HFNC was withdrawn immediately after closure of the surgical wound. The patient’s postoperative course was uneventful. The chest tube was removed on postoperative day 3. After rehabilitation, the patient was discharged on postoperative day 17. Performance status at discharge was 2 according to the Eastern Cooperative Oncology Group Performance Status, which was the same as that before the onset of pneumothorax. Although the patient continues to require oxygen therapy, no recurrence of pneumothorax was observed for 7 months after surgery.

## Discussion

This case highlights two issues. First, non-intubated uniportal VATS for SSP with an air-locking port, continuous pleural suction, and HFNC achieved satisfactory intraoperative oxygenation in a patient with respiratory dysfunction. Second, the intrapleural space was feasible for surgical manipulation without surgical pneumothorax in non-intubated VATS even when supplied with oxygen at a high flow rate using an HFNC.

Non-intubated uniportal VATS for SSP with an air-locking port, continuous pleural suction, and HFNC achieved satisfactory intraoperative oxygenation in a patient with respiratory dysfunction. In non-intubated VATS, the intrapleural space for surgical manipulation is usually obtained using surgical pneumothorax [5, 6]. However, because surgical pneumothorax involves collapse of the operative lung, it easily induces hypoxia [1, 2, 4], especially in patients with respiratory dysfunction. Indeed, as our group previously reported, with a conventional wound retractor, surgeons often need to interrupt surgical manipulation due to hypoxia and cover the wound with the palm while waiting for the expansion of the lung and recovery of oxygenation [2]. Our plan in the current case was to improve oxygenation by minimizing surgical pneumothorax and maintaining bilateral ventilation. An air-locking port reduces the inflow of air into the thorax. Pleural suction reduces the intrapleural pressure. HFNC provides positive pressure to the airway [7] and improves oxygenation [8]. These devices cooperate to counteract surgical pneumothorax and lung collapse. As a result, satisfactory oxygenation was achieved with moderate expansion of the operative lung. Air-locking ports have previously been used in VATS for pneumothorax. Some groups used air-locking trocars with small diameters in non-intubated multiportal VATS for spontaneous pneumothorax to control the inflation level of the lung [3, 9]. Other groups used a port for single-incision surgery in uniportal VATS with endotracheal intubation under general anesthesia [10–12]. To the best of our knowledge, the present case report is the first to describe non-intubated uniportal VATS for pneumothorax with an air-locking port to minimize surgical pneumothorax.

The intrapleural space was feasible for surgical manipulation without surgical pneumothorax in non-intubated VATS even when supplied with oxygen at a high flow rate using an HFNC. HFNC has been used in non-intubated VATS [1, 4, 13–16]. However, regardless of whether the flow rate was low (10–30 L/min) [4, 13, 14] or high (50–70 L/min) [15, 16], surgical pneumothorax was routinely introduced to obtain sufficient space for surgical manipulation. A higher flow rate expands the operative lung [4, 14]. In the current case, the expansion of the operative lung was incomplete despite the high flow rate of 60 L/min and lack of surgical pneumothorax, leaving the intrapleural space feasible for surgical manipulation. Incomplete expansion can be explained by the low compliance of the lung due to pulmonary fibrosis and air leakage. Our experience suggests that the administration of oxygen at a high flow rate via an HFNC without surgical pneumothorax can be acceptable in non-intubated VATS for pneumothorax secondary to restrictive lung disease.

The combination of an air-locking port, continuous pleural suction, and HFNC may facilitate the identification of the air-leak point in non-intubated VATS for SSP. Identification of the leak point is sometimes difficult in non-intubated VATS for SSP [2, 17]. Previous studies reported that sucking air from the pleural cavity [17] and positive airway pressure applied using manually assisted mask ventilation [18] are effective in identifying the leak point in non-intubated VATS. These attempts enlarge the pressure gradient across the visceral pleura, which drives air leakage and facilitates the identification of the leak point. In the current case, the reduction in intrapleural pressure using an air-locking port and continuous pleural suction and elevation of the airway pressure using HFNC may have contributed to the identification of the leak point. Thus, in addition to better oxygenation, our strategy using an air-locking port, continuous pleural suction, and HFNC in non-intubated uniportal VATS for pneumothorax may be beneficial in identifying the air-leak point.

The effect of an air-locking port and pleural suction in non-intubated VATS with the HFNC might be limited. Theoretically, the port and suction prevent collapse of the operative lung in collaboration with the HFNC. Because the three methods were used throughout surgery, it is difficult to determine the contribution of each method to satisfactory oxygenation and the successful termination of air leakage in this case. From the perspective of efficiency and cost effectiveness, the application of the air-locking port and pleural suction may seem redundant and expensive in the presence of the HFNC. It is possible that the surgery could be completed without them. However, incomplete expansion of the operative lung may have required administration of a higher fraction of inspired oxygen, which is a potential risk factor for postoperative acute exacerbation of idiopathic pulmonary fibrosis [19]. Patients with worse respiratory function than that of our patient may substantially benefit from an air-locking port and pleural suction. If an air-locking port is used, the intrapleural air may be suctioned through a connector for insufflation tubing on the port without a chest tube. The role of an air-locking port and suction in non-intubated VATS with HFNC should be examined in further studies.

## Conclusions

Non-intubated uniportal VATS for SSP with an air-locking port, continuous pleural suction, and HFNC may achieve satisfactory intraoperative oxygenation in patients with respiratory dysfunction. The intrapleural space can be feasible for surgical manipulation without surgical pneumothorax in non-intubated VATS even when supplied with oxygen at a high flow rate using an HFNC. Further research should be conducted to assess the usefulness of our strategy.

## Data Availability

Data supporting the conclusions are included in the article.

## Abbreviations

VATS: Video-assisted thoracic surgery
SSP: Secondary spontaneous pneumothorax
HFNC: High-flow nasal cannula
